# Memory B-cells are enriched in the blood of patients with acute Buruli ulcer disease: a prospective observational study

**DOI:** 10.1186/s12879-023-08370-1

**Published:** 2023-06-12

**Authors:** Jonathan Kofi Adjei, Wilfred Aniagyei, Ernest Adankwah, Julia Seyfarth, Ertan Mayatepek, Daniel Antwi Berko, Nancy Ackam, Max Efui Annani-Akollor, Samuel Asamoah Sakyi, Yaw Ampem Amoako, Dorcas Owusu, Marc Jacobsen, Richard Odame Phillips

**Affiliations:** 1 Kumasi Centre for Collaborative Research in Tropical Medicine, Kumasi, Ghana; 2 Department of Medical Diagnostics, College of Health Sciences, Kwame Nkrumah University of Science and Technology (KNUST), Kumasi, Ghana; 3 Department of General Pediatrics, Neonatology and Pediatric Cardiology, Medical Faculty, University Hospital Düsseldorf, Heinrich-Heine-University, Düsseldorf, Germany; 4 Department of Molecular Medicine, School of Medicine and Dentistry, College of Health Sciences, Kwame Nkrumah University of Science and Technology (KNUST), Kumasi, Ghana; 5 Department of Medicine, School of Medicine and Dentistry, College of Health Sciences, Kwame Nkrumah University of Science and Technology (KNUST), Kumasi, Ghana

**Keywords:** B-cell subsets, Buruli ulcer, *Mycobacterium ulcerans*, Biomarker

## Abstract

**Background:**

Buruli ulcer disease (BUD) caused by *Mycobacterium (M.) ulcerans* is characterized by necrotic skin lesions. As for other mycobacterial infections, e.g., tuberculosis, the immune response is important for host protection. B-cells may play a role in antimycobacterial immunity but studies characterizing the B-cell repertoire and memory generation in BUD and during the course of treatment are scarce.

**Methods:**

We investigated the adaptive immune cell repertoire in children with BUD and healthy matched controls by flow cytometry. Analyses prior to treatment, also in a study group of patients with tuberculosis, as well as three time points during BUD treatment (i.e., week 8, 16, and 32) were performed. In addition, BUD disease severity as well as treatment response were analysed for association with B-cell repertoire differences.

**Results:**

Children with BUD had comparable total B- and T-cell proportions but differed largely in B-cell subsets. Memory B-cell (B _mem_) proportions were higher in children with BUD whereas regulatory B-cell (B _reg_) proportions were lower as compared to healthy controls and tuberculosis patients. Lower naïve (B _naïve_) and higher transitional B-cell (B _trans_) proportions characterized children with BUD in comparison with tuberculosis patients. Under treatment, B _mem_ proportions decreased significantly whereas proportions of B _reg_ and B _naive_ increased concomitantly in children with BUD. Also, we found significant correlation between lesion size and B _mem_ as well as B _reg_. However, we did not detect associations between treatment efficacy and B-cell proportions.

**Conclusions:**

These results suggest a role of B-cell subsets in the immune response against *M. ulcerans*. Furthermore, changes in B-cell subset proportions may be used as markers for treatment monitoring in BUD.

**Supplementary Information:**

The online version contains supplementary material available at 10.1186/s12879-023-08370-1.

## Background

Buruli ulcer disease (BUD) is a necrotizing skin disease caused by *Mycobacterium ulcerans (M. ulcerans)*. The disease has been reported in over 33 countries worldwide with most cases from Africa. BUD ranks third only to tuberculosis and leprosy globally when considering mycobacterial diseases with significant public health impact. However, in endemic countries like Ghana and Cote d’Ivoire, it is second to tuberculosis [1, 2]. BUD can affect all age groups but in Africa, children aged five to fifteen years are mostly affected [3]. The clinical forms of BUD lesions vary significantly between the nodule, plaque, oedema and the late presenting disfiguring ulcers that occur when medical intervention is delayed. BUD lesions are categorized into 3 groups based on the size of lesion; category I- lesions less than 5 cm in diameter, category II- lesions between 5 and 15 cm in diameter and category III- lesions greater than 15 cm, or multiple or oedematous lesions [4].

Mycolactone is one possible factor underlying the interindividual variability in clinical presentation of affected patients. Unlike other mycobacterial organisms, *M. ulcerans* has a plasmid (i.e. pMUM) that codes for the synthesis of the lipid toxin mycolactone, the main virulence factor for *M. ulcerans* [5]. It has an analgesic effect, resulting in painless and indolent lesions. Furthermore, mycolactone has cytotoxic and immunosuppressive properties that dampen the local immune system while causing systemic immune disruption, which is responsible for the lack of inflammation seen in the early stages of the disease [6–10]. There is initial evidence that immunomodulation plays a role in BUD and we found immunopathology features of *M. ulcerans* specific T-cells in children with BUD previously [11].

Before 2004, surgical intervention was the mainstay of BUD management. Currently, management involves the administration of antibiotics, wound dressing and skin grafting or debridement and physiotherapy especially when lesions develop close to a joint. Although antibiotic therapy has made BUD management easier and with improved outcomes, the high degree of variability in time to healing among affected persons remains a challenge [12, 13]. While some researchers hypothesize that high mycolactone concentration is the underlying factor for slow healing times in lesions with high bacteria load when compared to similar lesions with relatively low bacteria load [14], others highlight genetic predispositions as accounting for the differences in the observed healing times [15]. Biomarkers associated with improved or adverse healing outcome to guide clinical decisions are highly needed.

Initial studies investigating biomarkers of treatment response looked at the effect of mycolactone on cell mediated immunity because of its role in the pathogenesis of BUD [16–19]. This focus on cell mediated immunity is understandable because of its well-established role in controlling intracellular mycobacterial organisms. However, histopathological findings in BUD lesions show the presence of acid-fast bacilli within the extracellular space [20], highlighting the possible role of humoral immunity in *M. ulcerans* disease control. B-cells may play a role in antimycobacterial immunity but studies characterizing the B-cell subsets in BUD and during the course of treatment are limited. The identification of such biomarkers of treatment response within the humoral immunity repertoire will be a useful guide for effective clinical management of BUD.

We have investigated the B-cell repertoire in children with BUD and healthy matched controls and changes that occur during antimycobacterial treatment. In addition, patients with tuberculosis (TB) were included to characterize BUD specific B-cell features as compared to another mycobacterial disease.

## Methods

### Study population, recruitment and selection criteria

This study was a prospective observational study. Between September 2014 and February 2016, patients with BUD were recruited at the Agogo Presbyterian Hospital in the Asante Akim North District, in the middle forest belt of Ashanti region of Ghana where there is high incidence of BUD [1].

A child or adolescent was recruited when the presenting lesion was consistent with the WHO clinical disease definition for BUD and diagnosis later confirmed by *M. ulcerans* IS2404 PCR. Up to 50% of all BUD cases in Africa are diagnosed in children below the age of 17 years [21, 22] with age being a significant factor for the clinical presentation of the disease [23]. Participants had to be ≤ 17 years of age, to be recruited into the study. Individuals with a history of BUD, leprosy, tuberculosis or HIV were excluded. Further, individuals with a history of recent antibiotic use or liver or kidney diseases were also excluded. A control group of age- and gender matched healthy contacts was recruited from siblings, relatives or household contacts of participants. TB patients selected based on clinical presentation, suggestive x-ray images, and positive immune test but without microbiological proof and yet to start any anti-tuberculosis therapy were recruited from within the community to serve as secondary controls. All participants were asked about their presenting active BUD disease, family history of BUD exposure, their previous medical history, household demographics and previous treatments.

### Diagnosis and treatment

Clinical BUD cases were confirmed by PCR for the *IS2404* repeat sequence specific for *M. ulcerans* [24]. Lesions were classified by their form: nodule, oedema, plaque or ulcer. The Aranz Medical Silhouette Camera (Aranz Medical, New Zealand) was used to measure the widest diameter in cm, the short diameter in cm, and the surface area of a lesion in cm^2^ (to take into account differences in the shape of lesions). Haematological parameters were assessed using the Sysmex XS-800i system (Sysmex).

Patients with BUD received a standard treatment regime with 15 mg/kg streptomycin and 10 mg/kg rifampicin daily for 8 weeks, based on recommended by the WHO [25]. Patients presented every two weeks during antibiotic treatment and monthly subsequently for monitoring of healing progress until complete healing.

### Immunological assays and flow cytometry

Up to 10 mL of venous blood was collected into heparinised blood collection tubes (BD Biosciences) between 9am and 12noon, prior initiation of antibiotic treatment. Blood samples were transported to the laboratory based in Kumasi and immediately processed (in less than 6 h after blood has been taken) [26, 27]. Surface staining using monoclonal antibodies and analyses by flow cytometry were performed to identify T-, B-cells as described [28]. In brief, the stains were performed in whole blood (100 µl) diluted with RPMI 1640 (100 µl) after centrifugation and discarding the supernatants. Following incubation with the fluorochrome-conjugated antibodies for 30 min at 4 °C, red blood cells were lysed using a red blood cell lysis buffer (Roche) according to manufacturers’ instructions. The remaining leucocytes were then fixed for 15 min at room temperature using Cytofix Solution (Biolegend). Stained white blood cells were acquired on a BD Accuri™ C6 Flow Cytometer (BD Biosciences) and analysed using FlowJo v10 (TreeStar).

Following anti-human antibodies were used for flow cytometric analysis: PerCP-Cy5.5.-conjugated CD3 (HIT3a), FITC- conjugated CD19, PE- conjugated CD1d, PerCP-Cy5.5 – conjugated CD24 and APC – conjugated CD38 All staining panels were evaluated using fluorescence minus one and unstained controls.

### Statistical analyses

Categorical associations were analysed with the chi-square test. The one -way ANOVA was used to analyse the haematology parameters between the groups because they were normally distributed (according to the Shapiro-Wilk test). Since remaining data were not normally distributed (according to the Shapiro-Wilk test) non-parametric test were applied. The Mann-Whitney *U* test was used for all pairwise comparisons (e.g. BUD patients vs. healthy contacts, Baseline vs. Week 8). Correlations were tested using Spearman’s rho analysis. Statistical analyses were performed using SPSS© v23 (IBM Corp.) and were taken as significant if p ≤ 0.05. Graphical illustration was done using Graphpad v8.0 (GraphPad Software, Inc.).

Lesion types were separated into groups based on their lesion form: pre-ulcerative forms (nodule/oedema/plaque) and ulcerative forms. Patients were also separated into two groups based on the time to healing following the start of antibiotic treatment, using a cut-off of 84 days (based on the median healing time = 84 days, range 14–231 days).

## Results

### Participant demographics and clinical characteristics

BUD patients (n = 52) with a median age of 8 years (range 1.5–17) and healthy community contacts (n = 29) (median age 7.0 years; range 3.0 − 15 years) were enrolled in this study. In addition, patients with tuberculosis (n = 37) (median age 5.0 years; range 1.0–16 years) were recruited. There was no significant difference in age and gender. The neutrophil and lymphocyte counts were higher and lower respectively in BUD patients compared to TB patients. There were no significant differences in the other haematology parameters between the study groups (Table 1). However, there were minor differences in corpuscular volume (MCV) values, platelets and eosinophil counts between the study groups but these did not reach statistical significance (Table 1). The clinical characteristics of the participants with BUD are shown in Table 2. Lesion sizes ranged from 2.3 to 206.2 cm^2^ among the BUD patients. All but two lesions were classified as less than category III lesions. The majority (42.3%) of patients presented with nodules.

**Table 1:** Participant Demographics

Parameter	BU-C	BU-P	TB-P	p-value
N	29	52	37	0.3581
Age (Range)	7(3–15)	8(1.5–17)	5(1–16)	
Sex				0.3866
Female	15 (51.7)	21 (40.4)	20 (54.1)	
Male	14 (48.3)	31 (59.6)	17 (45.9)	
BCG Scar	29	51*	37	0.0017
Absent	3 (10.3)	17 (33.3)	2 (5.4)	
Present	26 (89.7)	34 (66.7)	35 (94.6)	
Haematologic Parameters N**** (mean ± SEM)	**28**	**42**	**37**	
WBC	6.04 ± 0.53	7.58 ± 0.49	6.72 ± 0.57	0.1466
RBC	4.30 ± 0.17	4.27 ± 0.09	4.29 ± 0.12	0.9896
HB	11.37 ± 0.45	10.90 ± 0.19	10.53 ± 0.35	0.2277
HCT	32.63 ± 1.43	33.34 ± 0.71	34.36 ± 1.26	0.5761
MCV	76.05 ± 1.30	78.32 ± 1.06	80.21 ± 1.96	0.1845
PLT	239.8 ± 17.9	244.0 ± 21.5	269.8 ± 21.3	0.5598
Neutrophil	32.16 ± 2.09	36.93 ± 2.28	29.06 ± 2.44	0.0463
Lymphocyte	46.83 ± 2.56	39.96 ± 2.43	48.50 ± 2.47	0.0316
Monocyte	12.53 ± 1.38	11.04 ± 0.76	14.34 ± 1.48	0.1228
Eosinophil	6.30 ± 1.33	6.92 ± 0.95	4.70 ± 0.98	0.2894
Basophil	3.20 ± 0.76	2.58 ± 1.00	3.50 ± 1.04	0.7855

BU-C = Healthy contacts of BUD patients.

BU-P = BUD patients.

TB-P = TB patients,

*1 patient with unknown status.

**2 patients with unknown status.

***3 patients with unknown status.

****results unavailable for 1 healthy contact and 10 BUD patients.

**Table 2:** Clinical characteristics of patients with BUD

Clinical characteristics	n (%)
**Lesion Type**	
Ulcer	17 (32.7)
Nodule	22 (42.3)
Plaque	11 (21.2)
Oedema	2 (3.8)
**Lesion Site**	
Upper limb	18 (34.6)
Lower limb	28 (53.8)
**Other	6 (11.5)
**Time to Healing**	
Median (range) in days	84 (14–231)
Fast ≤ 84	19
Slow > 84	18

**Lesions on abdomen (n = 1), back (n = 1), thorax (n = 1), buttocks/perineum (n = 2), and head/neck (n = 1).

### Similar total B-cell proportions but differences in B-cell subsets between patients with BUD and controls

**Fig. 1:**
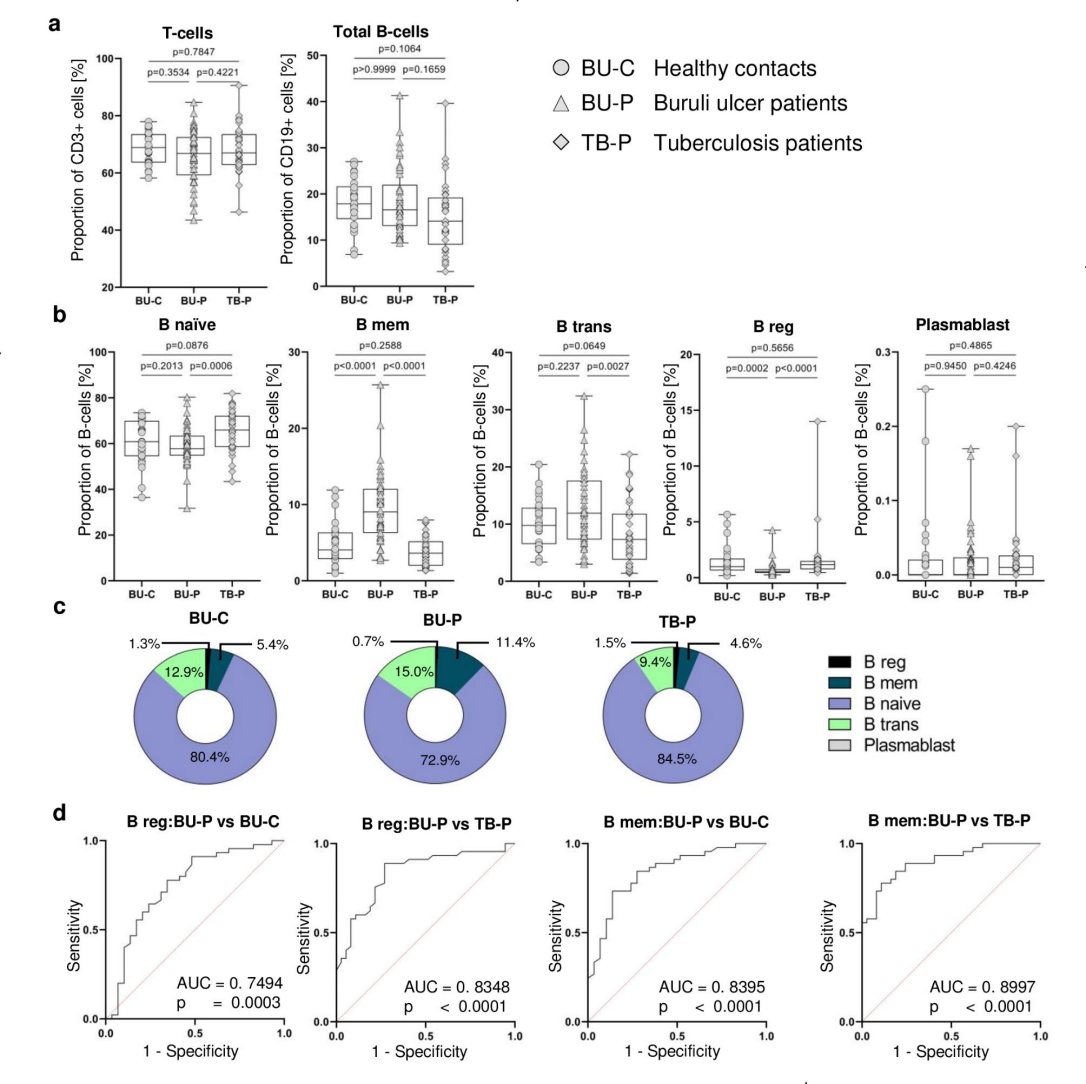
B-cell subset proportions in children with BUD and control groups. **(a)** The percentage proportion of total T-cells (CD3+) and B-cells (CD19+) in lymphocytic cells (**b**) The percentage proportion of B-Cell subsets (B _naive_, B _mem_, B _trans_, B _reg_, and plasmablasts). **(c)** Donut graphs depicting the relative proportions of B-cell subsets (B _mem_, B _reg_, B _naive_, B _trans_) in total B-cells of the participants within the 3 study groups **(d)** ROC analysis for discriminating BUD patients and contacts, and also BUD patients and TB patients using B _mem_ and B _reg_

Initially we compared total T-cell and B-cell proportions between the study groups. No significant differences in T-cell proportions between BUD patients and their community contacts were detected, and also TB patients had similar proportions of T-cells (Fig. 1a). Likewise, for total B-cell proportions, there were no significant differences between BUD patients and their community contacts or TB patients. Community contacts of BUD patients had a tendency of higher B-cell proportions as compared to TB patients (p = 0.1064).

Next, B-cell subsets were quantified and compared among the study population. The gating procedure is provided [see Additional File 1]. In contrast to total B-cell distribution we found marked differences in B-cell subsets (i.e., B _naïve_, B _trans_, B _mem_, and B _reg_) between the study groups (Fig. 1b). B _reg_ proportions were significantly lower in BUD patients when compared to their community contacts (p = 0.0002) and TB patients (p < 0.0001). B _reg_ proportion also could differentiate between BUD patients and community contacts (AUC = 0.7494; p = 0.0003) and between BUD patients and TB patients (AUC = 0.8348; p < 0.0001) as shown in Fig. 1d. Interestingly, B _mem_ proportions were significantly higher in BUD patients compared to their community contacts (p < 0.0001) and TB patients (p < 0.0001). Also, B _mem_ proportion differentiated between BUD patients and community contacts (AUC = 0.8395; p < 0.0001) and between BUD patients and TB patients (AUC = 0.8997; p < 0.0001) as shown Fig. 1d. B _trans_ proportion was significantly higher in BUD patients compared to TB patients (p = 0.0027) but did not differ between BUD patients and their community contacts (p = 0.2237), and between TB patients and community contacts (p = 0.0649). We further observed that B _naive_ proportion was significantly lower in BUD patients compared to TB patients (p = 0.0006) but did not differ between BUD patients and their community contacts (p = 0.2013), and between TB patients and community contacts of BU patients (p = 0.0876). These results suggested changes in the distribution of B-cell subsets towards higher B _trans_ and B _mem_ proportions in patients with BUD. This is visualized as donut pie charts for the study groups (Fig. 1c).

### B-cell subset proportions normalize in BUD patients during antibiotic treatment

**Fig. 2:**
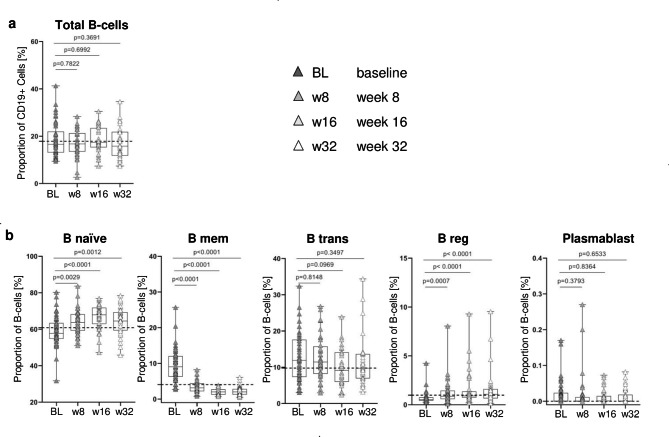
B-cell subset proportions during treatment of children with BUD. (**a)** The percentage proportion of B-cells (CD19+) in lymphocytic cells. (**b**) The proportions of B-cell subsets (i.e., B _naive_, B _mem_, B _trans_, B _reg_, and plasmablasts) for BUD patients at Baseline (BL), week 8 (w8), week 16 (w16) and week 32 (w32). The dashed lines correspond to the median mark of B-cell subsets measured in healthy contacts

Since differences in B-cell proportions could reflect immunopathology in untreated patients with BUD, we next compared total B-cell proportion and B-cell subsets in BUD patients before (baseline; BL), during (week (w)8 and w16 after treatment onset) and after treatment (w32) (Fig. 2). The total B-cell proportion in gated lymphocytic cells [see Addition file 1] did not differ significantly as therapy progressed (Fig. 2a). In contrast, B-cell subsets changed markedly during treatment with B _naive_ and B _reg_ proportions increasing markedly (Fig. 2b). This was significant between BL and all time points during and after treatment (B _naïve_: BL vs. w8, p = 0.0029; BL vs. w16, p < 0.0001; BL vs. w32, p = 0.0012) (B _reg_: BL vs. w8, p = 0.0007; BL vs. w16, p < 0.0001; BL vs. w32, p < 0.0001). Notably, the proportion of B _mem_ concomitantly decreased (BL vs. all time points, p < 0.0001). B _mem_, which made up a median value of 9.04 prior to treatment decreased towards 2.00 after treatment. This was comparable to BUD contacts (dotted line) and, hence, can be interpreted as normalization of B-cell distribution after successful treatment. There were no significant changes in the proportions of plasmablasts or B _trans_ during treatment.

### The BUD lesion size – but not ulceration or healing time – was associated with differential B-cell subset distribution

**Fig. 3:**
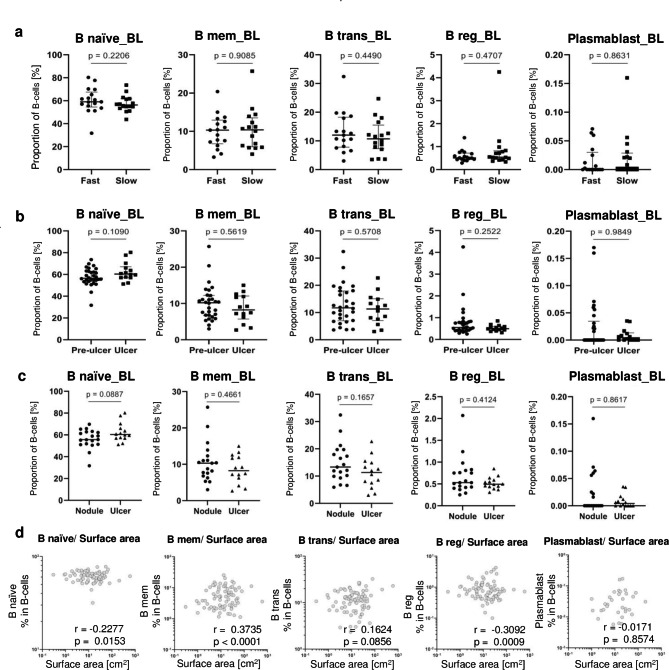
BUD lesion types, area size, and healing time analyzed for B-cell subset proportions. B-Cell subsets (B _naive_, B _mem_, B _trans_, B _reg_, and plasmablasts) in total B-cells from Buruli ulcer patients at baseline for **(a)** Fast Healers versus Slow Healers and **(b)** pre-ulcerative versus ulcerative lesions **(c)** nodules versus ulcers **(d)**The Spearman’s sign rank correlation between the surface area measurement of BUD lesions and proportions of B-cells

Changes in B-cell subset proportions during treatment rendered applicability of B-cell subsets for monitoring treatment response possible. Hence, we investigated if B-cell subset differences at BL are predictive for the time period of healing. To address this question, based on the BUD patients who had their date of healing recorded, 37 of patients were classified as either fast-healers (healing time ≤ 84 days) or slow healers (healing time > 84 days) under therapy as shown in Table 2 and compared for the different B-cell subsets at baseline. No significant differences were detected for the proportions of B _naïve_, B _trans_, B _reg_ and B _mem_ as well as for plasmablasts between fast healers and slow healers (Fig. 3a). Next, we compared BUD lesion type [i.e., pre-ulcer vs. ulcer and nodules vs. ulcers (Fig. 3b and c)] or the lesion size (Fig. 3d) for differences in B-cell subsets. No significant differences were detected between patients presenting with pre-ulcerative or ulcerative lesion (Fig. 3b) or between nodules and ulcers (Fig. 3c) in the B-cell subset proportions. In contrast, we detected a positive correlation of B _mem_ proportions with lesion size (r = 0.37; p < 0.0001) as well as negative correlations for B _naive_ and B _reg_ proportions (r=-0.23; p = 0.015; r=-0.31; p = 0.0009, respectively; Fig. 3d). We concluded that the BUD lesion size but not ulceration or healing time were associated with high B _mem_ and low B _naive_/B _reg_ proportions.

## Discussion

Here, we present findings on the first study to analyse the B-cell repertoire between patients with BUD and controls while at the same time performing a prospective evaluation of the B-cell subset proportions in the context of BUD treatment progression.

B _mem_ proportions were enriched and could discriminate BUD patients from TB patients and healthy household contacts. Additionally, a significant decline in B _mem_ proportion was observed during BUD therapy. These findings were in keeping with other studies that demonstrated B _mem_ proportion can be used to differentiate patients from healthy individuals in various diseases [29] [30]. The reason for differential B _mem_/B _naïve_/B _reg_ proportions in the peripheral blood is unclear. Systemic effects of mycolactone in BUD may be an explanation for the changes in the immune cell distribution. It is also known that in tuberculosis systemic pathology results in changes in immune cell distribution. [28] The reason for higher B mem may be insufficient migration of this subset into the affected skin tissue due to systemic effects of mycolactone. Indeed, this is consistent with the knowledge that mycolactone causes a selective suppression effect on immune cells which is reversed after antibiotic treatment [31–33]. Further studies need to be conducted to investigate the role of mycolactone in B _reg_ differentiation and recruitment of B _mem_. Alternatively, the lower B _reg_ concentration in the blood of BUD patients compared to the other groups may indicate systemic immune efforts to boost inflammation for healing. The increase in the B _reg_ proportion during treatment is in line with the modulation of systemic inflammatory response within the context of healing. Although we were not able to determine the specificity of B-cell subsets in BUD patients, changes in the proportions during treatment suggested specificity for *M. ulcerans* of affected B-cells [10]. Notably, the increased B _mem_ proportion observed in BUD patients could result from priming by previous exposure to *M. tuberculosis* antigens within the environment because the study area is co-endemic for TB and BUD. Therefore, subsequent exposure to *M. ulcerans* antigens would elicit a heightened memory response. The higher B _mem_ proportion could also have resulted from the chronicity of *M. ulcerans* infections. This warrants further investigations to elucidate the role of B _mem_ in BUD in co-endemic areas.

To evaluate whether the proportions of B-cell subsets could distinguish the different clinical forms of BUD, we compared BUD patients with pre-ulcerative and ulcerative lesion types. None of the subsets including B _reg_ and B _mem_ could differentiate between the two groups. At present, the WHO-recommended treatment regimen for BUD is rifampicin and clarithromycin. Despite the 90% treatment success, the healing process varies significantly among patients. While healing of BUD lesions begin immediately after antibiotic initiation in some, others commence several weeks after treatment initiation. Healing times for BUD lesions also vary considerably from 4 weeks to 48 weeks [13]. Moreover, paradoxical reactions, a phenomenon identified in BUD may complicate the healing process for between 13% and 20% of patients [14]. Given that healing is an immunological process, identification of immune biomarkers for predicting healing in BUD would be essential for effective management of the disease.

In this study, healing was observed between 14 and 231 days (median 84) which is consistent with the range observed by Sarfo et al., 2010 [34]. Comparing ‘slow’ and ‘fast’ healers revealed that none of the B-cell subsets differed between the two groups. This is likely due to other immunological factors as reported in Nausch et al., 2017 [11] and other factors such as nutritional status [35]. This study is unable to provide data on paradoxical reactions and requires further investigations.

While this study has made some novel findings, we recognize that it is limited in that it focuses on children and adolescents. Notwithstanding, majority of BUD cases in Africa occur in children and hence this age group would benefit most from the potential biomarkers identified here. We recommend that the B _mem_ and B _reg_ are tested in older BUD patients who are more likely to develop severe forms of the disease. Furthermore, since one of the main features of *M. ulcerans* infection is its local consequences in skin (with analgesia, cytotoxicity and immunomodulation), future studies that focus on the skin-local B-cell subsets distribution are recommended.

## Conclusions

In conclusion, B _mem_ and B _reg_ differ between BUD patients and healthy contacts as well as between BUD patients and TB patients. Also, B _mem_ and B _reg_ differed between baseline and as therapy progressed. Hence B _mem_ and B _reg_ are potential biomarkers for monitoring treatment progression in BUD.

## Electronic supplementary material

Below is the link to the electronic supplementary material.

Supplementary Material 1

## Data Availability

All data generated or analysed during this study are included in this published article and its additional file.

## List of abbreviations

BUD: Buruli ulcer disease
*M. ulcerans*: *Mycobacterium ulcerans*
BU-C: healthy contacts of Buruli ulcer patients
BU-P: Buruli ulcer patients
TB-P: Tuberculosis patients
B _mem_: Memory B-cells
B reg: Regulatory B-cells
B naïve: Naïve B-cells
B _trans_: Transitional B-cells
WHO: World Health Organisation
TB: tuberculosis
PCR: Polymerase chain reaction
WBC: White blood cell count
RBC: Red blood cell count
HB: Haemoglobin concentration
HCT: Haematocrit
MCV: Mean corpuscular volume
PLT: Platelet count
n: number
BL: Baseline
w: week
AUC: Area under curve
