# Supplementary figures and images for: Memory B-cells are enriched in the blood of patients with acute Buruli ulcer disease: a prospective observational study
Source: BMC Infect Dis. 2023 Jun 12;23:393. doi: 10.1186/s12879-023-08370-1 (PMC10259001; doi:10.1186/s12879-023-08370-1)

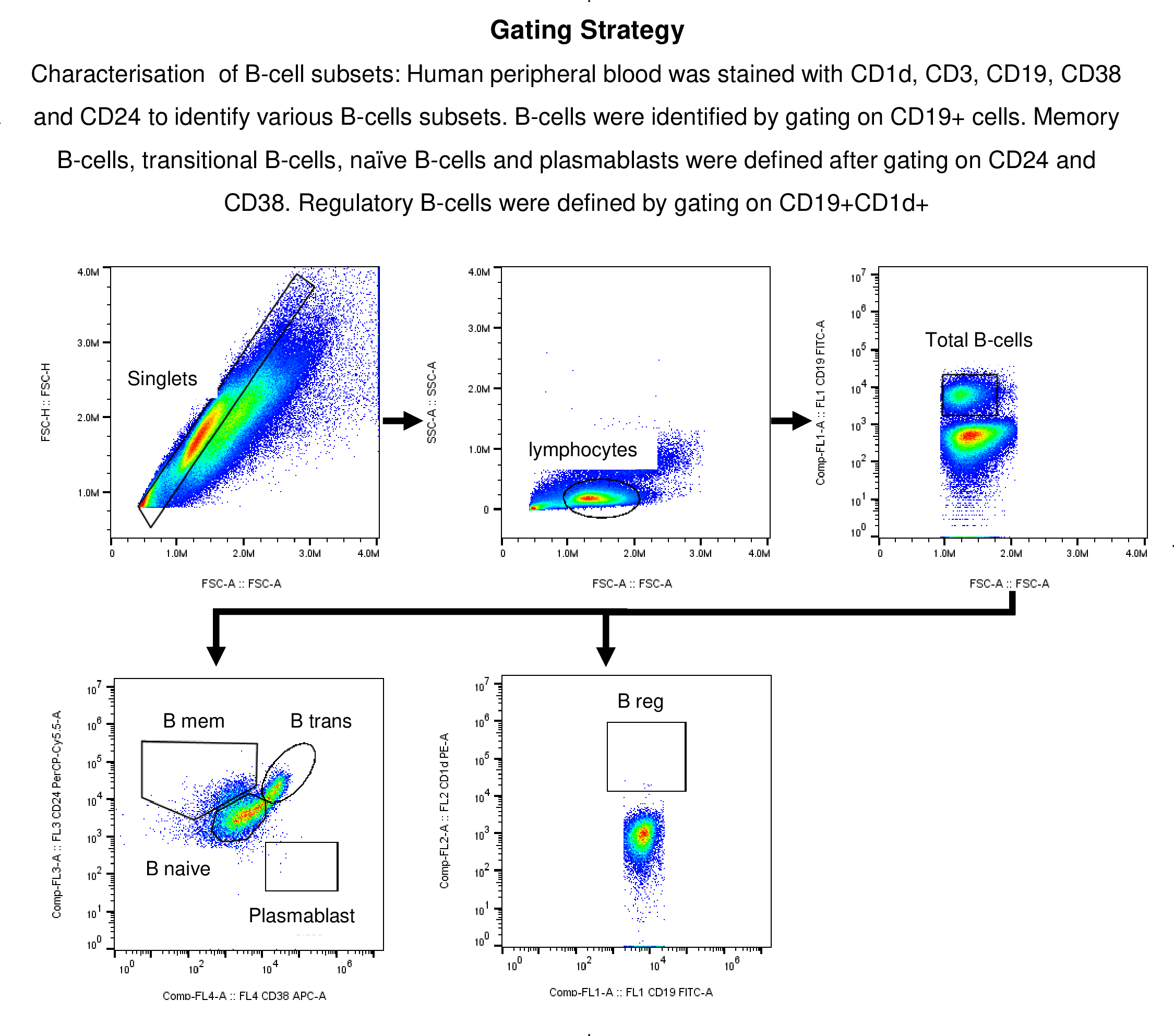

Supplement: Supplementary file 1 — Supplementary Material 1 [file 12879_2023_8370_MOESM1_ESM.tif]
